# A VvWRKY8-salicylic acid pathway amplification loop confers resistance to *Plasmopara viticola* in grapevine

**DOI:** 10.1186/s43897-026-00233-y

**Published:** 2026-08-05

**Authors:** Huimin Huang, Junjie Qu, Jiaqi Liu, Wei Wu, Jiang Lu

**Affiliations:** 1 Center for Viticulture and Enology, School of Agriculture and Biology, Shanghai Jiao Tong University, Shanghai, China; 2 Chongqing Research Institute, Shanghai Jiao Tong University, Chongqing, China; 3 Guangxi Crop Genetic Improvement and Biotechnology Key Lab, Guangxi Academy of Agricultural Sciences, Nanning, China

**Keywords:** Grapevine, *Plasmopara viticola*, Transcriptional regulation, Salicylic acid (SA)

## Abstract

To defend against pathogen invasion, plants deploy a variety of strategies, among which salicylic acid (SA), a key plant defense hormone, plays crucial roles in enhancing host resistance to biotrophic and semibiotrophic microbes. Although numerous studies elucidated mechanisms of the SA signaling pathway, many questions remain. In this study, we show that the group IIc WRKY transcription factor VvWRKY8 is involved in grape (*Vitis vinifera*) defense responses to the oomycete pathogen *Plasmopara viticola,* as indicated by transcriptome analyses. VvWRKY8 increases the expression of defense-related genes and SA accumulation, thereby promoting grape resistance to *P. viticola*. Further analyses reveal that VvWRKY8 is recruited to the promoters of *VvCBP60g* and *VvSARD1*, which encode two key regulators of SA biosynthesis, and upregulates their transcription*.* Moreover, VvWRKY8 transcription is induced by SA and by two bZIP transcription factors, VvTGA2a and VvTGA2b, which cooperate with the SA receptors VvNPR1 and VvNPR3 to modulate the expression of SA-responsive genes. Collectively, our results indicate that a positive feedback loop involving VvWRKY8 and the SA pathway components VvCBP60g, VvSARD1, and VvTGA2a/2b functions in response to *P. viticola* attack in grapevine.

**Supplementary Information:**

The online version contains supplementary material available at 10.1186/s43897-026-00233-y.

## Core

VvWRKY8 directly upregulates the expression of *VvCBP60g* and *VvSARD1*, which encode two key transcription factors in SA biosynthesis, thereby enhancing grapevine resistance to *Plasmopara viticola*. In turn, *VvWRKY8* expression is directly regulated by VvTGA2a and VvTGA2b, components of the SA receptor complex, indicating that *VvWRKY8* is a SA-responsive gene. VvWRKY8 functions as an immune-related protein in grapevine and represents an important component of SA signaling.

## Gene & accession numbers

Sequence data involved in this article can be found in the GenBank or Phytozome (https://phytozome-next.jgi.doe.gov/) data libraries with following accession numbers.

*VvWRKY8* (PQ058695/VIT_201s0010g03930), *VvCBP60g* (PQ133196/VIT_214s0060g01470), *VvSARD1* (PQ133197/VIT_217s0000g03380), *VvTGA2a* (PQ133198/VIT_213s0084g00660), *VvTGA2b* (PQ133199/VIT_208s0007g05170), *VvNPR1* (PQ133200/VIT_211s0016g01990), *VvNPR3* (PQ133201/VIT_210s0042g01250), *VvPR1* (VIT_203s0088g00810), *VvPR2* (VIT_208s0007g06030), *VvPR4* (VIT_14s0081g00030), *VvPR5* (VIT_203s0038g02170), *VvICS1* (VIT_201s0010g00900), *proVvWRKY8* (PQ133203), *proVvCBP60g* (PQ133204), and *proVvSARD1* (PQ133205).

## Introduction

Plants are challenged by a variety of pathogens, including bacteria, fungi, and oomycetes,and have evolved numerous defense strategies for survival (Jones et al. 2016; Jones and Dangl 2006). Pathogen-associated molecular patterns (PAMPs) are recognized by pattern-recognition receptors (PRRs) on the plasma membrane, activating pattern-triggered immunity (PTI) that limits microbial attack (Jones and Dangl 2006; Dangl et al. 2013). To overcome host defenses, microbes secrete effectors that dampen PTI, resulting in effector-triggered susceptibility (ETS) (Jones and Dangl 2006). Plant hosts, in turn, recognize these effectors directly or indirectly through nucleotide-binding leucine-rich repeat receptors (NLRs), initiating effector-triggered immunity (ETI) (Jones and Dangl 2006; Laflamme et al. 2020). Although PTI and ETI are activated by distinct mechanisms, they share similar downstream immune signaling pathways, including reactive oxygen species (ROS) production, Ca^2+^ influx, mitogen-activated protein kinase (MAPK) activation, and transcriptional reprogramming (Yuan et al. 2021; Tena 2021). ETI is more robust and is often accompanied by the hypersensitive response (HR) (Yuan et al. 2021; Tena 2021). Accordingly, effectors can impair both PTI and ETI, thereby increasing plant susceptibility (Ruiz-Bedoya et al. 2023). To further restrict pathogen spread, localized acquired resistance (LAR) occurs in the cells surrounding the infected area to inhibit pathogen propagation (Jacob et al. 2023). Unknown immunogenic molecules or pro-immune-response factors are synthesized and released from infected cells and perceived by neighboring cells, triggering LAR, and establishing a quarantine that prevents microbial spread (Jacob et al. 2023; Coster et al. 1999).

The salicylic acid (SA) signaling pathway is a powerful defense mechanism against pathogen invasion (Yuan et al. 2021). In *Arabidopsisthaliana*, chorismate, the precursor of SA, is converted to isochorismate in plastids by ICS1 (Isochorismate Synthase 1) or ICS2 (Mercado-Blanco et al. 2001; Wildermuth et al. 2001). Isochorismate is transported to the cytosol by EDS5 (Enhanced Disease Susceptibility 5) (Nawrath et al. 2002), modified with glutamate by PBS3 (AvrPphB Susceptible 3) (Rekhter et al. 2019), and finally converted to SA either spontaneously or with the assistance of EPS1 (Enhanced Pseudomonas Susceptibility 1) (Torrens-Spence et al. 2019; Zheng et al. 2009). SA levels increase dramatically after pathogen infection, following PRRs recognize pathogens and transmit signals into the cytoplasm (Peng et al. 2021). Two calmodulin-binding protein 60-like (CBP60) family transcription factors, SARD1 (Systemic Acquired Resistance Deficient 1) and CBP60g, are activated and recruited to the promoters of *ICS1*, *EDS5,* and *PBS3*, thereby upregulating their expression (Zhang et al. 2010; Wang et al. 2011; Sun et al. 2015). TGA2/5/6 (TGACG-binding factor 2/5/6) interacts with NPR1 (Nonexpresser of Pathogenesis-Related Genes 1) and other components, to form a transcriptional coactivator complex (Zhang et al. 1999; Wu et al. 2012). When SA is sensed by NPR1, TGA2/5/6 and NPR1 provide DNA-binding activity and transcriptional activation activities, respectively, promoting the expressions of SA-induced genes such as *PR* (Pathogenesis-Related) genes (Rochon et al. 2006; Zhang et al. 2003). A positive feedback mechanism further amplifies SA signaling by increasing active NPR1and upregulating *CBP60g* and *SARD1* expression (Peng et al. 2021; Ding et al. 2018; Ishihama et al. 2021).

SA is well known for its central role in systemic acquired resistance (SAR). CBP60g and SARD1 indirectlyactivate the production of N-hydroxypipecolic acid (NHP), a mobile SAR signal. When immune signals are transmitted to distal tissues by NHP, NHP in turn induces *CBP60g* and *SARD1* expression (Ding et al. 2018; Chen et al. 2018; Hartmann et al. 2018). SA also contributes to PTI and ETI. Exogenous SA induces the expression of PTI marker genes, including *FLS2, CERK1, MAPKKK5,* and *CPK6*, as well as ETI marker genes, including *EDS1, PAD4, SAG101,* and *NDR1 *(Ding et al. 2018; Falk et al. 1999; Feys et al. 2001). The SA-deficient mutant *sid2-2 (SA-deficient 2–2,* generated by mutation of *ICS1)* shows reduced flg22-induced resistance to *Pst* DC3000 *hrcC-* compared with the wild type (Tsuda et al. 2008). In addition, *sid2-1*, *npr1-1, npr1-2, npr1-3, npr4-4D,* and the double mutant *npr1-1 npr4-4D,* are more susceptible to *Pto* DC3000 *AvrRpt2* or *Pto* DC3000 *AvrRps4*, avirulent bacterial strains that trigger ETI (Liu et al. 2020; Zhang and Li 2019; Chen et al. 2021a, b, c).

WRKY transcription factors (TFs) play pivotal roles in regulating plant tolerance to biotic stresses (Wang et al. 2023a, b). In *A. thaliana*, the WRKY family consists of 72 members characterized by a conserved WRKYGQK motif at the N-terminus of the DNA-binding domain. (Eulgem et al. 2000; Rushton et al. 1995). WRKY TFs recognize and bind the W-box element [(C/T)TGAC(C/T)] in the promoters of target genes, thereby positively or negatively regulating transcription (Javed and Gao 2023). Several WRKY TFs participate in SA signaling and modulate plant immunity. For instance, AtWRKY70 binds the promoter of *AtSARD1* and dynamically regulates its expression through phosphorylation-dependent mechanisms with or without *Pst* DC3000 infection (Zhou et al. 2018; Liu et al. 2021). AtWRKY55 is recruited to the promoters of *AtICS1* and *AtPBS3* and upregulates their expression, resulting in SA accumulation (Wang et al. 2020). MdWRKY46 activates the expression of *MdPBS3.1* and enhances resistance to *Botryosphaeria dothidea* in apple (Zhao et al. 2019). In rice, OsWRKY67 promotes blast and bacterial blight resistance by directly upregulating the expression of *OsPR1a* and *OsPR10* (Liu et al. 2018). Overexpression of CsWRKY50 confers tolerance to downy mildew by enhancing the expression of *CsICS1* and *CsPR* genes in cucumber (Luan et al. 2019).

Although the mechanisms of SA signaling have been extensively explored in recent years, many issues remain unresolved. For example, which elements complete the positive feedback amplification of SA signaling? Moreover, many WRKY TFs regulate plant immunity via SA signaling, but their molecular mechanisms remain unclear, particularly in crops. Downy mildew caused by the obligate biotrophic oomycete *Plasmopara viticola,* leads to significant losses in viticulture worldwide each year. To date, less is known about the molecular mechanisms underlying grapevine resistance to *P. viticola*, which has hindered the breeding of resistant cultivars for controlling grape downy mildew (Koledenkova et al. 2022). Here, we demonstrate that a WRKY family member in *Vitis vinifera*, VvWRKY8, modulates grape immunity against *P. viticola* by directly regulating the expression of *VvCBP60g* and *VvSARD1,* and by being activated by VvTGA2s as a SA-responsive gene, thereby forming positive feedback loop.

## Results

### Transcriptome analysis suggests that VvWRKY8 functions in the interaction between *V. vinifera* and* P. viticola*

**Fig. 1:**
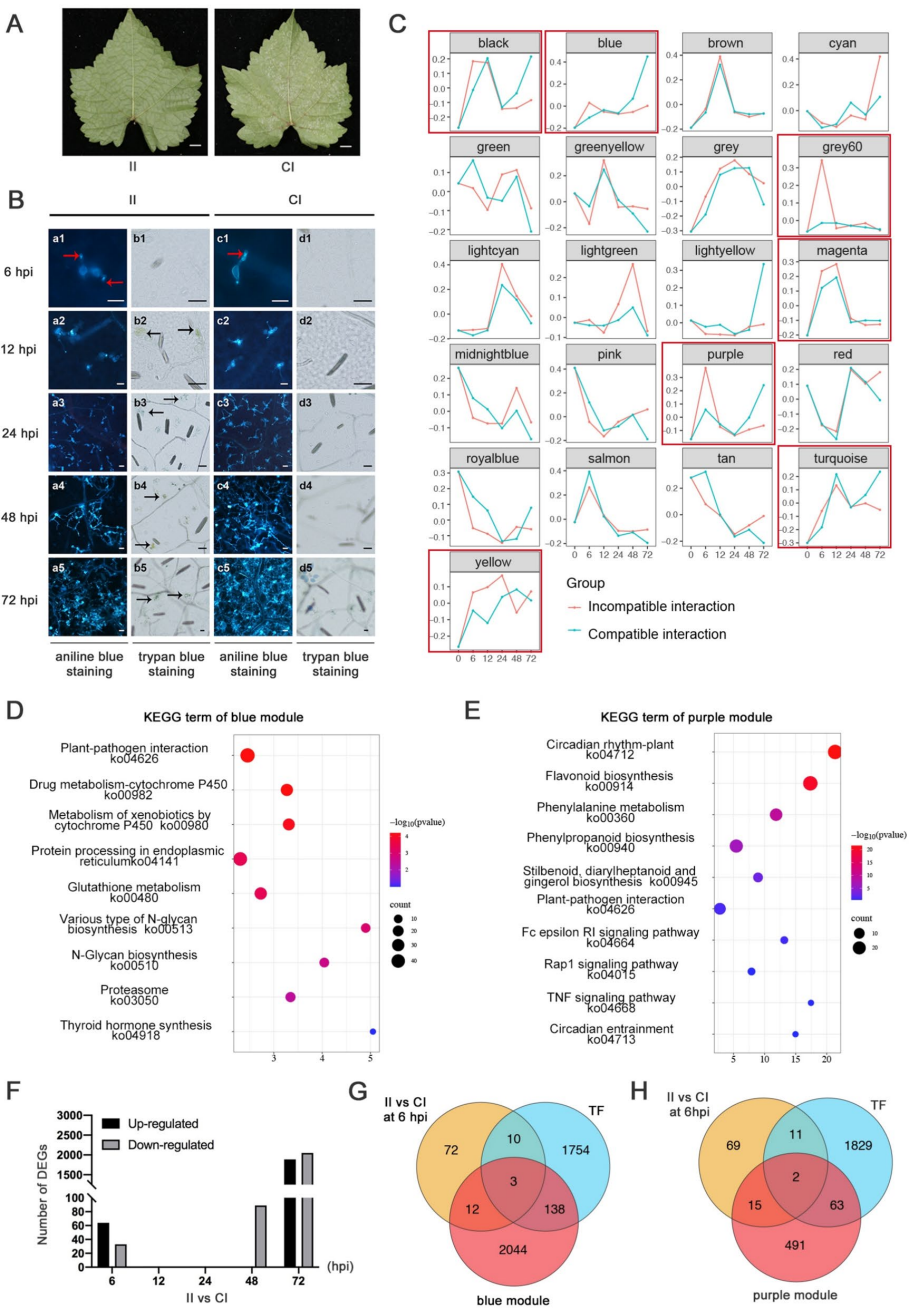
Transcriptome analysis of compatible interaction (CI) and incompatible interaction (II) in grapevine resistance to *P. viticola*. **A** Phenotypes of grape leaves inoculated with different *P. viticola* isolates. ‘Cabernet Sauvignon’ grape leaves were inoculated with *P. viticola* isolates *Pv*18005 (II) and *Pv*18002 (CI), respectively.Images were taken in 7 days after infection. Scale bars represent 1 cm. **B** Histochemical staining reveals the interaction processes between grape and two *P. viticola* isolates. Aniline blue and trypan blue staining assays were conducted at different time points after *P. viticola* infection of grape leaves. Red arrows indicate the haustoria of *P. viticola*, and black arrows indicate necrotic lesions on grape leaves. Scale bars represent 50 µm. **C** WGCNA analysis. The x-axis represents hours after inoculation, and the y-axis represents the module eigengene. **D** and **E** KEGG enrichment analyses of the blue module (**D**) and purple module (**E**). The x-axis represents the enrichment factor, and the y-axis represents the pathway. Dot size indicates gene number, and dot color indicates *p* value. **F** Numbers of DEGs between II and CI at each time point.** G** and **H** Venn diagrams showing the common genes among II vs. CI, TFs, and the blue module (**G**), and among II vs. CI, TFs and the purple module (**H**)

A previous study showed that almost all Chinese *P. viticola* isolates could infect susceptible Eurasian grapevine, and that only one isolate (*Pv*18005) failed to do so (Wu et al. 2025; Fig. S1A). In this study, leaves of the Eurasian grapevine cultivar ‘Cabernet Sauvignon’ exhibited distinct phenotypes upon inoculation with two *P. viticola* isolates. Pv18005 provoked a slight HR and its growth was limited, indicating an incompatible interaction(II), whereas Pv18002 showedhigher biomass and more successful infection, indicating a compatible interaction(CI) (Fig. 1A; Fig. S1B). Histochemical staining assays revealed that the haustoria of *P. viticola* form before 6 hours post inoculation (hpi), and necrotic lesions were observed from 12 hpi in II but were not observed even at 72 hpi in CI (Fig. 1B). These results suggest that *P. viticola* invasion and *V. vinifera* defense responses occur at an early stage of the interaction, before 12 hpi*.* To further investigate the molecular mechanisms underlying *V. vinifera* responses to *P. viticola*, II and CI samples were collected at each time point for transcriptome sequencing. A total of 20,076 genes were obtained after filtering raw reads to remove adapters and unknown or low-quality data, and aligning the remaining reads to the grapevine genome PN40024 (GenBank assembly accession No: GCA_000003745.2) (Data 1). In weighted gene co-expression network analysis (WGCNA), these genes were clustered into 21 modules based on their expression patterns. Based on the histochemical staining results, we focused on the black, blue, grey60, magenta, purple, turquoise and yellow modules, because genes in these 7 modules, were upregulated at 6 and 12 hpi compared with 0 hpi, and their expression levels were higher in II than in CI (Fig. 1C; Data 1). Kyoto Encyclopedia of Genes and Genomes (KEGG) analyse of these 7 modules showed that only the blue and purple modules were associated with plant immunity, as genes in these modules were categorized into plant-pathogen interaction or phytoalexin metabolism pathways (Fig. 1D and E; Data 2).The numbers of differentially expressed genes (DEGs) between 0 hpi and each time point within CI and II, as well as between CI and II at each time point, were analyzed (Fig. S1, C and D; Data 3 and 4). In II vs. CI, 97, 1, 1, 89, and 3,937 DEGs were identified at 6, 12, 24, 48 and 72 hpi respectively (Fig. 1F, Data 5). To identify key TFs regulating *V. vinifera* resistance to *P. viticola*, the 97 DEGs at 6 hpi in II vs. CI, 1,905 TF genes, and genes in the blue or purple modules were compared using Venn diagrams (Data 6). One MYB TF gene, one C2H2 TF gene, one ERF TF gene, and two WRKY TF genes were common to both comparisons (Fig. 1, G and H; Data 7). Motif enrichment analysis of genes in the blue and purple modules using the MEME Suite tool (https://meme-suite.org/meme/doc/download.html) showed that the WRKY TF-binding element W-box was highly enriched (Data 7 and 8). A transcriptional activity assay of the two WRKY TFs revealed that VvWRKY8 (VIT_201s0010g03930), but not VvWRKY12 (VIT_204s0069g00980), exhibited transcriptional activity (Fig. S1E). Therefore, we focused on VvWRKY8 and investigated its function in responses to *P. viticola*.

### Characteristics of VvWRKY8

**Fig. 2:**
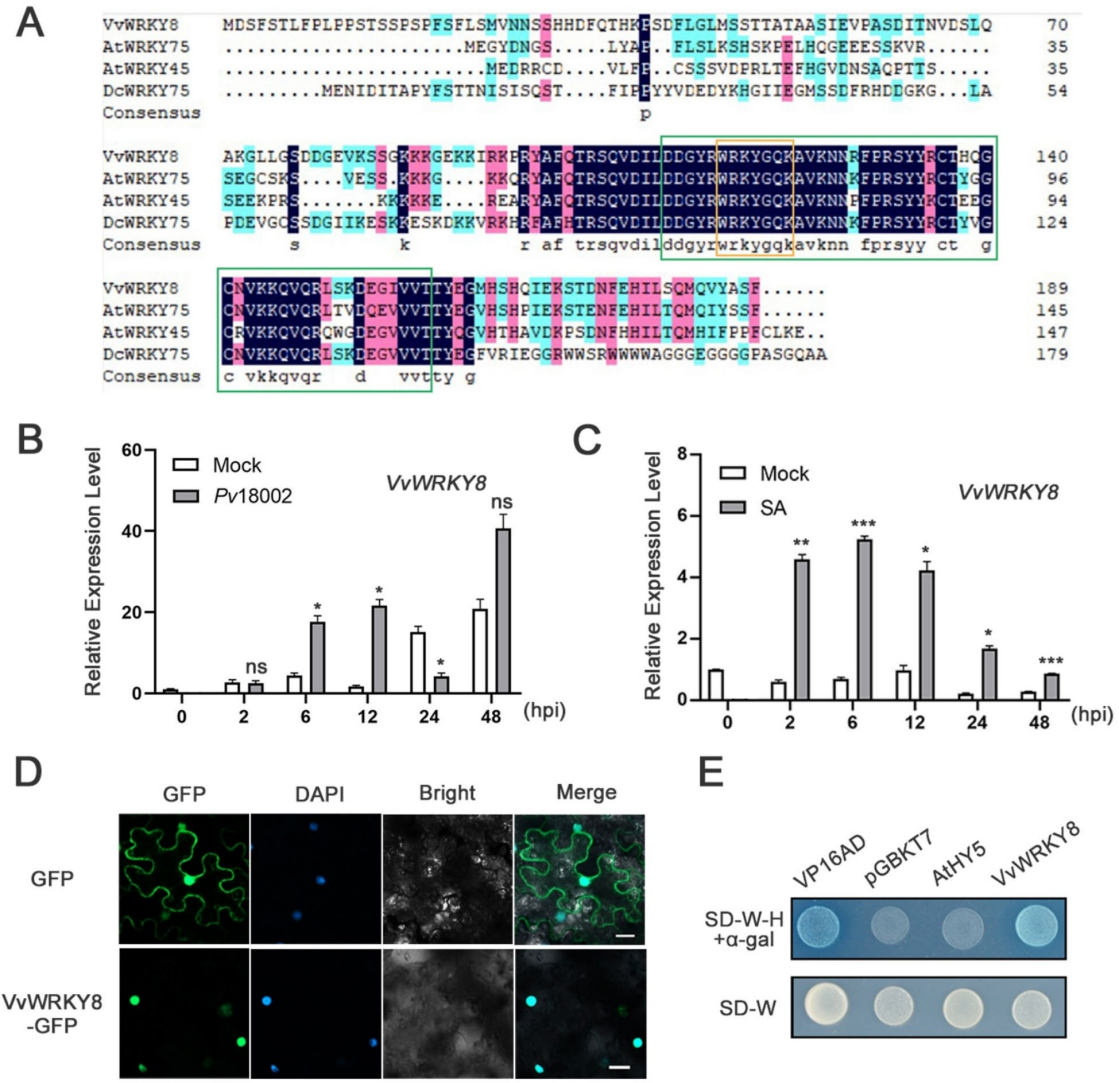
Expression patterns, subcellular localization, and transcriptional activity of VvWRKY8. **A** Alignment of the amino acid sequences of VvWRKY8 and its homologs. AtWRKY45 and AtWRKY75 are from *Arabidopsis thaliana*, and DcWRKY75 is from *Dianthus caryophyllus* L. Orange boxes indicate WRKY motifs, and green boxes indicate WRKY domains. **B** and **C** RT-qPCR-based transcriptional profiling of *VvWRKY8* in ‘Cabernet Sauvignon’ leaves following inoculation with *P. viticola* isolate *Pv*18002 (**B**) or treatment with 100 µM SA (**C**) at the indicated time points. *VvGAPDH* was used as an internal reference. Expression levels at 0 hpi under *P. viticola* (**B**) or SA (**C**) treatment were set to 1. Error bars represent the SE of three biological replicates. ns, no significant; *, *p* value < 0.05; **, *p* value < 0.01; ***, *p* value < 0.001 (two-way ANOVA and Sidak’s tests). **D** VvWRKY8 localizes to the nucleus. GFP or VvWRKY8-GFP was expressed in tobacco leaves via agroinfiltration, and fluorescence signals were examined by confocal microscopy 2 days after transformation. DAPI was injected into tobacco leaves before observation. Scale bar is 20 µm. **E** Analysis of VvWRKY8 transcriptional activity. Yeast strain AH109 was transformed with the indicated vectors. VP16AD was used as a positive control, whereas pGBKT7 and AtHY5 were used as negative controls. Two days after transformation, positive transformants were selected on SD/-Trp medium and transferred to SD/-Trp/-His medium supplemented with 80 mg/mL α-gal. Images were captured 2 days later

*VvWRKY8* encodes a group IIc WRKY family member, which is 189 amino acids in length with a typical WRKY domain at the C terminus (Fig. 2A). Multiple sequence alignment of VvWRKY8 and homologs from other species showed high conservation within the WRKY domains (Fig. 2A). Based on real-time quantitative PCR (RT-qPCR) analysis of *V. vinifera* following inoculation with the *P. viticola* isolate *Pv*18002, *VvWRKY8* expression peaked at 6 hpi, suggesting that *VvWRKY8* is involved in early immune responses during host–pathogen interactions. A second peak was observed at 48 hpi (Fig. 2B). Next, grape leaves were treated with 100 µM SA, and samples were collected at the indicated time points. RT-qPCR analysis showed that SA rapidly induced *VvWRKY8* expression compared with mock treatment (Fig. 2C). The induction of *VvPR1*, a representative SA-responsive gene, confirmed the reliability of this treatment (Fig. S2G). *VvWRKY6* (*VIT_217s0000g01280*) and *VvWRKY47* (*VIT_214s0068g01770*) are homologs of *VvWRKY8* in *V. vinifera* (Wang et al. 2014a, b), however, their expression was only slightly induced in both the transcriptome and SA treatment assays compared with *VvWRKY8* (Fig. S2, A to F; Fig. 2C). Therefore, we focused on *VvWRKY8* in subsequent analyses. To determine the subcellular localization of VvWRKY8, a recombinant VvWRKY8-GFP construct was transiently expressed in *Nicotiana benthamiana* via *Agrobacterium-*mediated transformation. Fluorescence signals were detected in the nucleus and overlapped with DAPI staining under confocal microscopy, indicating that VvWRKY8 is a nuclear protein (Fig. 2D). To assess the transcriptional activity of VvWRKY8, pGBKT7 fused with the *VvWRKY8* CDS was transformed into yeast strain AH109. Positive transformants were transferred to SD/-Trp/-His medium supplemented with α-gal. It had been reported that bZIP transcription factor AtHY5 lacks transcriptional activity, a fusion construct containing *AtHY5* and Herpes Simplex Virus VP16 activation domain (*VP16AD*) was included as described previously (Stracke et al. 2010; Chen et al 2021a,b, c). In this assay, VP16AD served as a positive control, whereas AtHY5 and pGBKT7 served as negative controls. Blue yeast clonies indicated that VvWRKY8 possesses transcriptional activity (Fig. 2E).

### VvWRKY8 confers resistance to *P. viticola* in *V. vinifera*

**Fig. 3:**
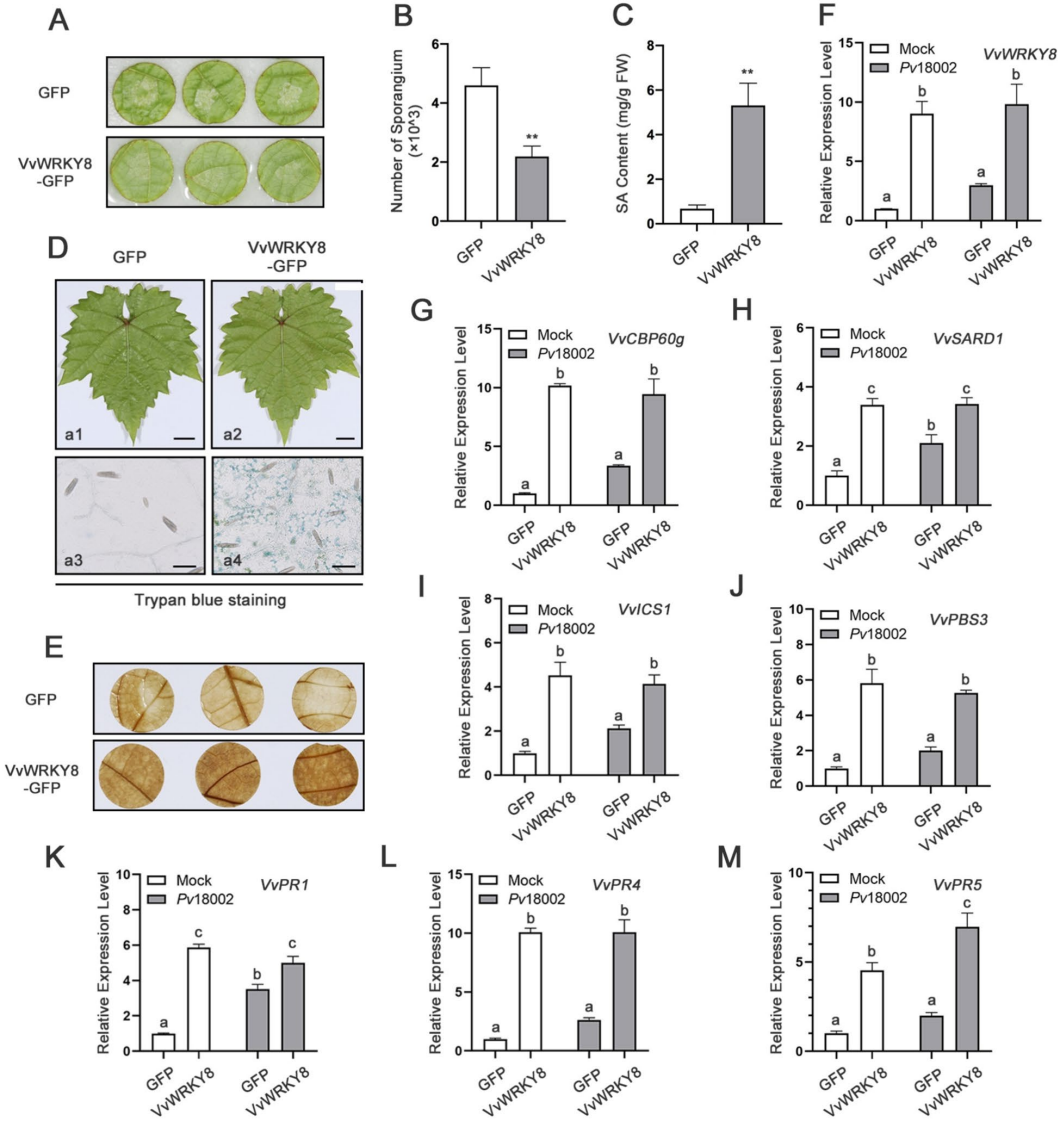
Transient expression of VvWRKY8 enhances resistance to *P. viticola* in ‘Thompson Seedless’ grape leaves. **A** Phenotypes of ‘Thompson Seedless’ grape leaf discs inoculated with *P. viticola*. Leaf discs expressing GFP or VvWRKY8-GFP were challenged with *P. viticola* 5 days after transformation. *P. viticola* growth was assessed 7 days post inoculation. **B** Number of sporangia as an indicator of *P. viticola* biomass. Sporangia of *P. viticola* on each leaf disc expressing GFP or VvWRKY8-GFP were counted 7 days post inoculation. Error bars represent SE of eight biological replicates (**, *p* value < 0.01, Student's t-test). Similar results were obtained in three independent experiments. **C** SA content of ‘Thompson Seedless’ grape leaves expressing GFP or VvWRKY8-GFP. Samples were collected 5 days after transformation. Total SA (free SA and SA glucoside) was measured by HPLC. Error bars represent SE of eight biological replicates (**, *p* value < 0.01, Student's t-test). Similar results were obtained in two independent experiments. **D** Transient expression of VvWRKY8-GFP triggers cell death in ‘Thompson Seedless’ grape leaves. Phenotypes of grape leaves expressing GFP or VvWRKY8-GFP are shown in a1 and a2, respectively. Trypan blue staining of dead cells is shown in a3 and a4. Scale bars, 1 cm (a1, a2) and 500 µm (a3, a4). **E** VvWRKY8 induces ROS accumulation in *V. vinifera*. Five days after transformation, grape leaf discs were stained with DAB. **F** to **M** RT-qPCR analysis of transcripts of *VvWRKY8* (**F**), *VvCBP60g* (**G**), *VvSARD1* (**H**), *VvICS1* (**I**), *VvPBS3* (**J**), *VvPR1* (**K**), *VvPR4* (**L**), and *VvPR5* (**M**) in ‘Thompson Seedless’ grape leaves. Samples were collected 5 days after transformation and then treated with ddH_2_O (Mock) or *P. viticola* isolate *Pv*18002. Expression levels were normalized to those in mocktreated GFP leaves, and *VvGAPDH* was used as the internal reference. Error bars represent the SE of four biological replicates. Different letters indicate significant differences (*p* > 0.05) based on two-way ANOVA followed by Tukey’s honestly significant difference (HSD) test

To determine the role of VvWRKY8 in *V. vinifera* responses to *P. viticola* attack, grape leaves transiently overexpressing GFP or the recombinant protein VvWRKY8-GFP via agroinfiltration were challenged with *P. viticola* isolate Pv18002 5 days after transformation, and *P. viticola* biomass was measured 7 days after inoculation. The transcription and translation levels of VvWRKY8-GFP were confirmed by RT-qPCR (Fig. S3B) and fluorescence signals (Fig. S3A), respectively. Compared with the GFP control, *P. viticola* growth was suppressed on VvWRKY8-overexpressing grape leaves (Fig. 3A). Consistently, the number of sporangia, used as an index of *P. viticola* biomass, was lower on grape leaves expressing VvWRKY8-GFP than on control leaves (Fig. 3B). DAB staining indicated that VvWRKY8 induced ROS accumulation (Fig. 3E). Transient expression of VvWRKY8-GFP also triggered cell death in grape leaves, which was confirmed by trypan blue staining (Fig. 3D). Total SA in infiltrated grape leaves was quantified by HPLC, and VvWRKY8 increased SA levels in grape (Fig. 3C). We next performed RT-qPCR to analyze the transcripts of *VvCBP60g*, *VvSARD1*, *VvICS1*, *VvPBS3*, *VvPR1*, *VvPR4*, *VvPR5*, and *VvWRKY8*. Overexpression of *VvWRKY8* induced the expression of all these genes (Fig. 3F to M). Together, these results indicate that VvWRKY8 enhances grape immunity and reduces susceptibility to *P. viticola*.

### VvWRKY8 directly upregulates *VvCBP60g* and *VvSARD1* expression

A key question is how VvWRKY8 mediates plant immune responses.Because *VvWRKY8* expression enhances SA accumulation (Fig. 3C), the expression patterns of *VvCBP60g* (blue module) and *VvSARD1* (purple module) during *P. viticola* infection are consistent with that of *VvWRKY8* (Fig. 2B; Fig. S3, A and B). In addition, W-box elements are present in the promoters of *VvCBP60g* and *VvSARD1* (Fig. 3A). We therefore hypothesized that VvWRKY8 acts as a transcriptional regulator of *VvCBP60g* and *VvSARD1.*

**Fig. 4:**
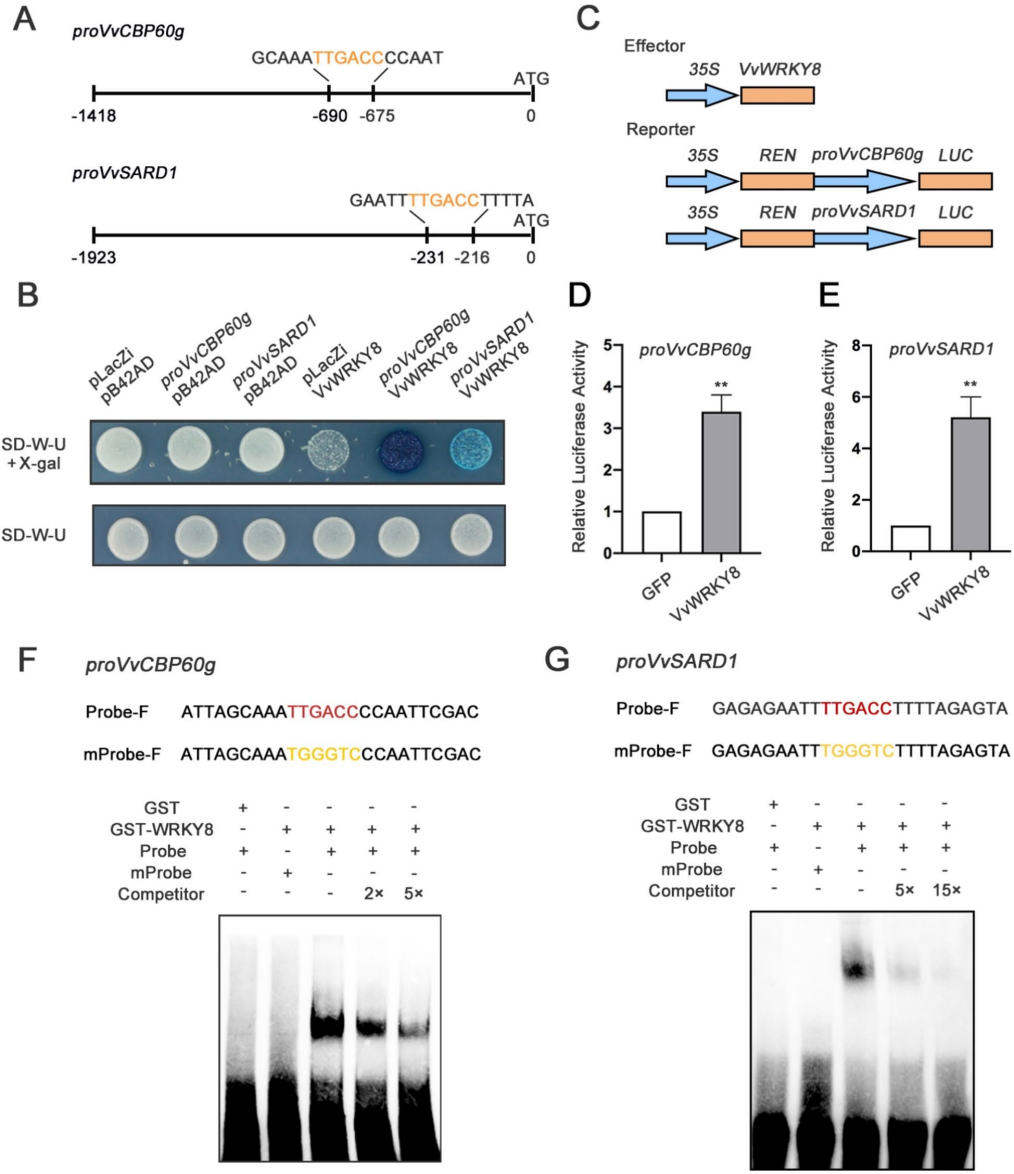
VvWRKY8 regulates *VvCBP60g* and *VvSARD1* expression. **A** Schematic representation of the promoter sequences of *VvCBP60g* and *VvSARD1*. **B** Y1H assays showing that VvWRKY8 binds to the promoters of *VvCBP60g* and *VvSARD1*. Transformants were grown on SD/-Trp/-Ura medium supplemented with 80 mg/mL X-gal. **C** Vector constructs used for dual-luciferase assays. The promoters of *VvCBP60g* and *VvSARD1* drive expression of the reporter gene firefly luciferase (*LUC*). **D** and **E** Dual-luciferase analyses showing VvWRKY8-dependent activation of *proVvCBP60g*::LUC and *proVvSARD1*::LUC. LUC activity was normalized to Renilla luciferase (REN) activity. The co-expression of the reporter and GFP was set to 1. Error bars indicate SE of six biological replicates. Statistical significance was determined by Student’s t-test (**, *p* value < 0.01). Similar results were obtained in three independent experiments. **F** and **G** EMSA showing interactions between VvWRKY8 and the promoters of *VvCBP60g* and *VvSARD1*. The sequences of biotin-labeled probes and mutant probes are shown. “mProbe” indicates a probe with mutated W-box. Unlabeled probe was used as a competitor, and 2 ×, 5 ×, and 15 × indicate the amount of competitor relative to the biotin-labeled probe

To determine whether *VvCBP60g* and *VvSARD1* are regulated by VvWRKY8, we performed yeast-one-hybrid (Y1H) assays. Yeast transformants harboring pB42AD-VvWRKY8 together with the reporter vectors pLacZi-pro*VvCBP60g* or pLacZi-pro*VvSARD1* turned blue on SD/-Trp/-Ura medium supplemented with 80 mg/mL X-gal, indicating that VvWRKY8 binds to the promoters of *VvCBP60g* and *VvSARD1* (Fig. 4B). We then performed an EMSA to confirm the direct binding of VvWRKY8 to these promoters. Biotin-labeled 25 bp probes containing W-box elements derived from the *VvCBP60g* and *VvSARD1* promoters were used (Fig. 4F and G). GST and recombinant GST-WRKY8 proteins were expressed in *E. coli* and purified. GST-WRKY8 shifted both biotin-labeled probes, supporting direct binding (Fig. 4F and G). In dual-luciferase assays, VvWRKY8 under the control of the CaMV 35S promoter was used as the effector, and the promoters of *VvCBP60g* and *VvSARD1* were fused to reporter constructs (Fig. 4C). VvWRKY8 expression increased LUC activity driven by both promoters compared with the control (Fig. 4D and E). Consistently, RT-qPCR assays showed that overexpression of *VvWRKY8* induced *VvCBP60g* and *VvSARD1* transcription in grape (Fig. 3G and H). Together, these results indicate that VvWRKY8 directly activates *VvCBP60g* and *VvSARD1* transcription.

### VvCBP60g and VvSARD1 promote *V. vinifera *resistance to *P. viticola*

**Fig. 5:**
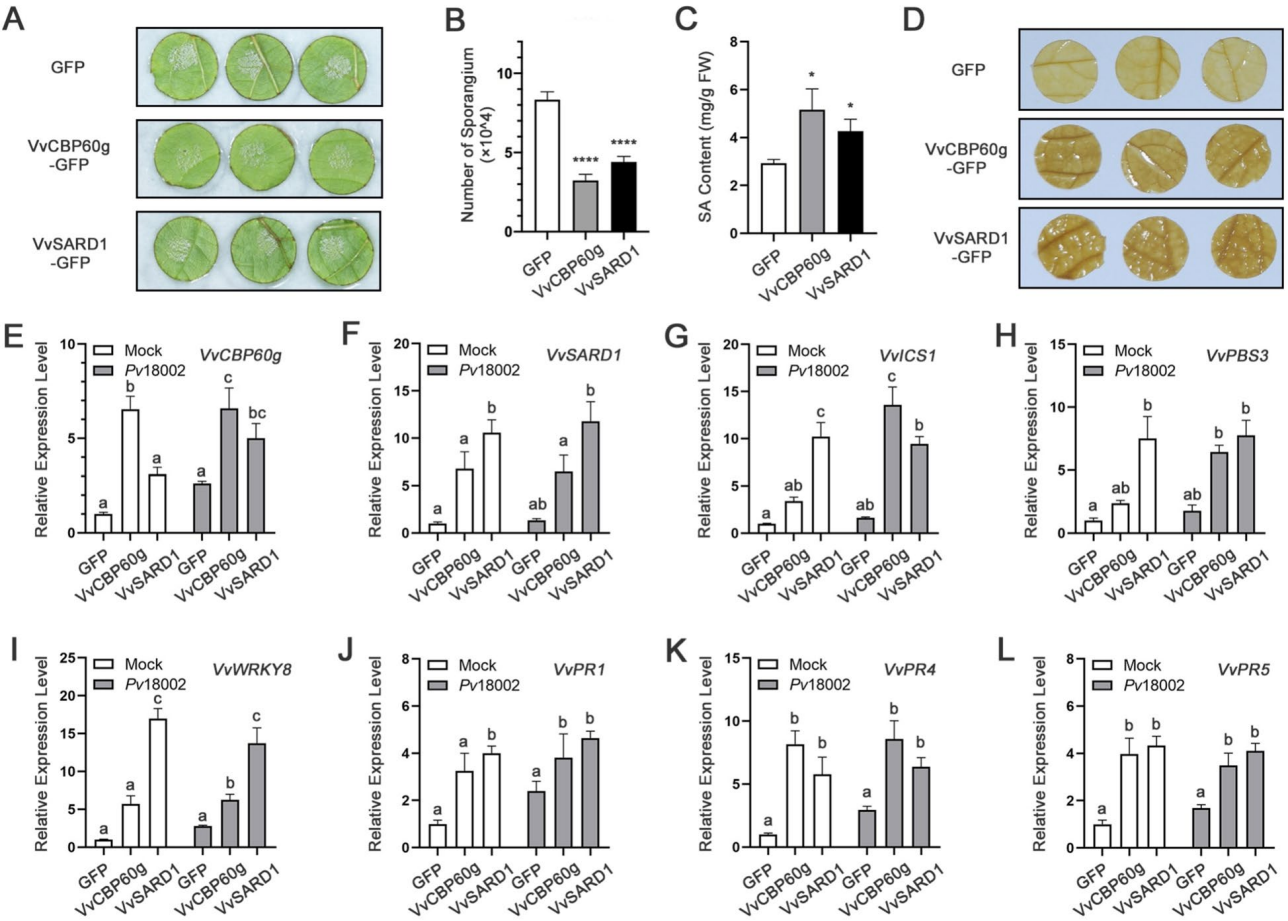
Transient expression of VvCBP60g and VvSARD1 confers resistance to *P. viticola* in *V. vinifera*. **A** *P. viticola* susceptibility assay. Five days after Agrobacterium-mediated transformation, grape leaf discs were inoculated with *P. viticola*. Images were captured at 7 days post inoculation. **B** Sporangia counting. Sporangia per leaf disc were quantified at 7 days post inoculation. Error bars indicate SE (*n* = 6). ****, *p* value < 0.0001 (Student's t-test). **C** SA quantification. Total SA levels in leaves expressing GFP, VvCBP60g-GFP, or VvSARD1-GFP were determined by HPLC-MS. Error bars indicate SE (*n* = 6). *, *p* value < 0.05 (Student's t-test). **D** DAB staining assay. Leaf discs expressing GFP, VvCBP60g-GFP, and VvSARD1-GFP were stained with DAB and decolorized after 24 h. **E** to **L** Transcript levels of *VvCBP60g* (**E**), *VvSARD1* (**F**), *VvICS1* (**G**), *VvPBS3* (**H**), *VvWRKY8* (**I**), *VvPR1* (**J**), *VvPR4* (**K**), and *VvPR5* (**L**) following overexpression of VvCBP60g or VvSARD1 in *V. vinifera*. At 5 days after transformation, samples were treated with ddH₂O or *P. viticola* for 12 h and then collected. *VvGAPDH* was used as the internal reference. Expression levels in the GFP control were set to 1. Error bars indicate SE (*n* = 4). Different letters indicate significant differences (*p* value < 0.05) based on two-way ANOVA followed by Tukey’s HSD test. Bars sharing at least one letter are not significantly different

VvCBP60g and VvSARD1, two key transcriptional regulators of SA biosynthetic genes, localized to the nucleus (Fig. S5D), and their transcription was induced following SA treatment (Fig. S5, A and B). To investigate the roles of VvCBP60g and VvSARD1 in grape defense against pathogens, *Agrobacterium* carrying the overexpression constructs pHB*-VvCBP60g-GFP* or pHB*-VvSARD1-GFP* was introduced into grape leaves by vacuum infiltration. Expression of the VvCBP60g-GFP and VvSARD1-GFP fusion proteins was confirmed by fluorescence (Fig. S5C). Five days after *Agrobacterium*-mediated transformation, leaves were excised into discs and inoculated with *P. viticola*. Compared with the GFP control, leaf discs expressing VvCBP60g-GFP or VvSARD1-GFP showed reduced *P. viticola* biomass and fewer sporangia, indicating decreased susceptibility to *P. viticola* in *V. vinifera* (Fig. 5A and B)*.* Transient expression of VvCBP60g and VvSARD1 also increased endogenous SA accumulation and ROS production (Fig. 5C and D), but did not trigger programmed cell death, as assessed by trypan blue staining (Fig. S5E). RT-qPCR analyses further showed that overexpression of *VvCBP60g* increased not only *VvCBP60g* transcript levels but also *VvSARD1* transcript levels, consistent with feedback amplification of SA signaling (Fig. 5E). A similar pattern was observed in samples overexpressing VvSARD1 (Fig. 5F). Overexpression of either VvCBP60g or VvSARD1 induced the transcription of SA-related genes, including *VvICS1*, *VvPBS3*, *VvWRKY8*, *VvPR1*, *VvPR4*, and *VvPR5* (Fig. 5G to L).

### The expression of *VvWRKY8* is activated by VvTGA2a and VvTGA2b

**Fig. 6:**
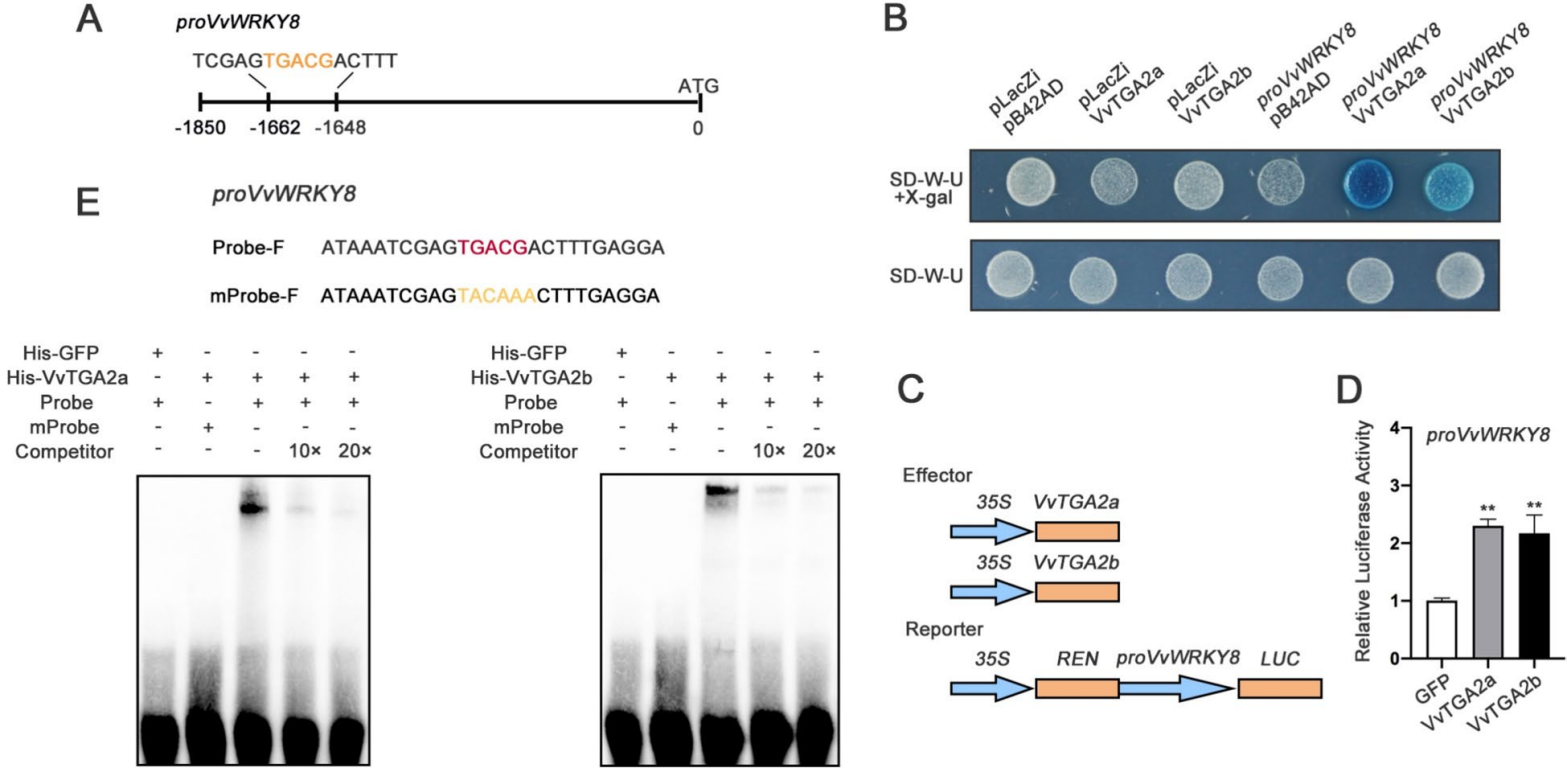
Both VvTGA2a and VvTGA2b bind to the promoter of *VvWRKY8* and activate its expression. **A** Schematic representation of the *VvWRKY8* promoter. **B** Y1H analysis of the interaction between VvTGA2a/2b and the *VvWRKY8* promoter. Yeast growth on SD/-Trp/-Ura selection medium supplemented with 80 mg/mL X-gal is shown. **C** Schematic diagrams of the effector and reporter constructs used in the dual-luciferase assay. The *VvWRKY8* promoter drives LUC expression, whereas 35S-driven *VvTGA2a* and *VvTGA2b* serve as effectors. **D** Relative luciferase activity (LUC/REN) of the indicated effectors and reporter in *N. benthamiana*. The activity of the reporter with *35S*::*GFP* was set to 1. Five biological replicates were performed, and significance was assessed by Student's t-test (**, *p* value < 0.01). The experiment was repeated four times and a representative result is shown. **E** EMSA analysis of VvTGA2a/2b binding to the *VvWRKY8* promoter. The probe is the biotin-labeled wild type TGACG box from the *VvWRKY8* promoter, “mProbe” is the biotin-labeled mutant probe, and the competitor is the unlabeled wild type probe

The SA-induced expression of *VvWRKY8* (Fig. 2C), the presence of a TGA-binding cis-element (TGACG motif) in the *VvWRKY8* promoter (Fig. 6A), and the upregulation of *VvWRKY8* transcription by *VvCBP60g* and *VvSARD1* (Fig. 5I) prompted us to investigate whether *VvWRKY8* is regulated by VvTGAs. In *A. thaliana*, AtTGA2, AtTGA5, and AtTGA6 regulate SA-induced *PR* gene expression in cooperation with AtNPR1 and AtNPR3 (Rochon et al. 2006; Zhang et al. 2003). We performed a BLAST search to identify *A. thaliana* AtTGA2 orthologs in *V. vinifera*. Phylogenetic analysis indicated that two *V. vinifera* proteins, designated VvTGA2a (VIT_213s0084g00660.2) and VvTGA2b (VIT_208s0007g05170.3), are orthologs of AtTGA2 (Fig. S6A).

**Fig. 7:**
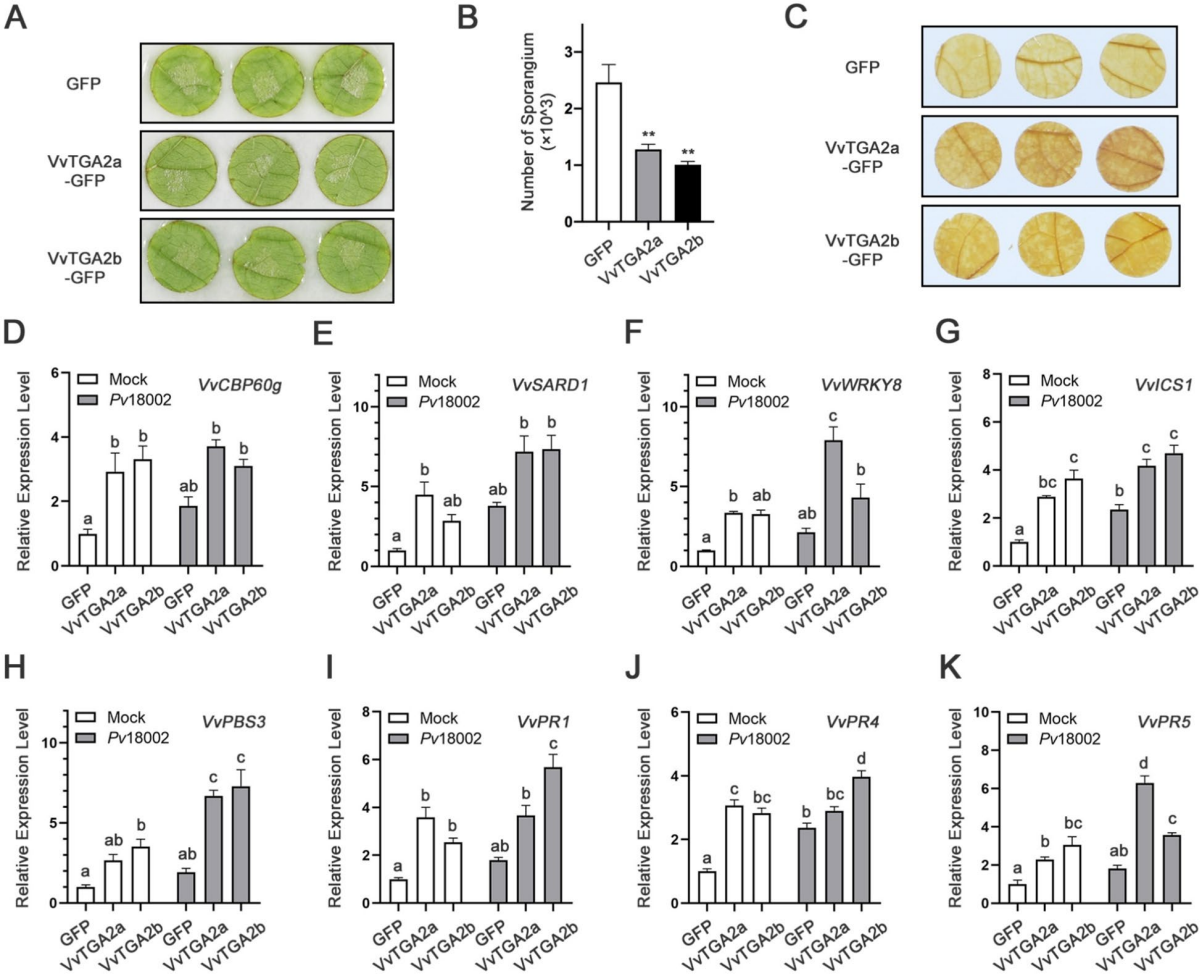
VvTGA2a and VvTGA2b enhance resistance to *P. viticola* in grapevine. **A** Phenotypes of grape leaf discs transiently expressing GFP, VvTGA2a or VvTGA2b and inoculated with *P. viticola*. Images were taken in 7 days after inoculation. **B** Sporangia counts at 7 days after inoculation. Error bars indicate SE (*n* = 6). **, p value < 0.01 (Student's t-test). **C** DAB staining showing ROS accumulation. **D** to **K** RT-qPCR assays showing transcript levels of *VvCBP60g* (**D**), *VvSARD1* (**E**), *VvWRKY8* (**F**), *VvICS1* (**G**), *VvPBS3* (**H**), *VvPR1* (**I**), *VvPR4* (**J**), and *VvPR5* (**K**). Transformed grape leaves were treated with ddH_2_O (Mock) or *P. viticola* isolate Pv18002 and sampled at 12 h. *VvGAPDH* served as the internal reference. Expression levels in Mock and GFP controls were set to 1. Error bars indicate SE (*n* = 4). Different letters denote significant differences (p value < 0.05) by two-way ANOVA followed by Tukey’s HSD test. Bars sharing letters are not significantly different

Consistent with Fig. 4, the interactions of VvTGA2a and VvTGA2b with the *VvWRKY8* promoter were detected by Y1H assays (Fig. 6B) and further confirmed by dual-luciferase assays (Fig. 6C and D; Fig. S6B) in vivo and by EMSA (Fig. 6E) in vitro. In grape leaves overexpressing VvTGA2a or VvTGA2b, *VvWRKY8* transcript levels increased, and these increases were further enhanced upon *P. viticola* treatment (Fig. 7F).

### VvTGA2a and VvTGA2b decrease *V. vinifera* susceptibility to *P. viticola*

Neither *P. viticola* infection nor SA treatment induced the transcription of *VvTGA2a* or *VvTGA2b* (Fig. S7). This suggests that post-transcriptional regulation may play a larger role in VvTGA2 activation. The bZIP transcription factors VvTGA2a and VvTGA2b displayed transcriptional activity and localized to the nucleus (Fig. S8, A and B). VvTGA2a and VvTGA2b interacted with the SA receptors VvNPR1 and VvNPR3 in vitro (Fig. S9A) and in vivo (Fig. S9B), resembling their Arabidopsis homolog AtTGA2.

VvTGA2a and VvTGA2b were transiently expressed in ‘Thompson Seedless’ grape leaves under the control of the double CaMV 35S promoter with a GFP tag. Their expression levels were evaluated by RT-qPCR (Fig. S9, C and D) and fluorescence detection (Fig. S9E). In *P. viticola* susceptibility assays and sporangia counts, VvTGA2a and VvTGA2b enhanced resistance to *P. viticola* (Fig. 7A and B). VvTGA2a and VvTGA2b also increased ROS accumulation, as indicated by DAB staining (Fig. 7C). RT-qPCR analyses further showed that transient expression of VvTGA2a and VvTGA2b modestly increased the transcript levels of their downstream target *VvWRKY8* and other SA-related genes, and these increases were further enhanced by *P. viticola* infection, suggesting that pathogen challenge may facilitate post-transcriptional regulation of VvTGA2 activity (Fig. 7D to K).

## Discussion

**Fig. 8:**
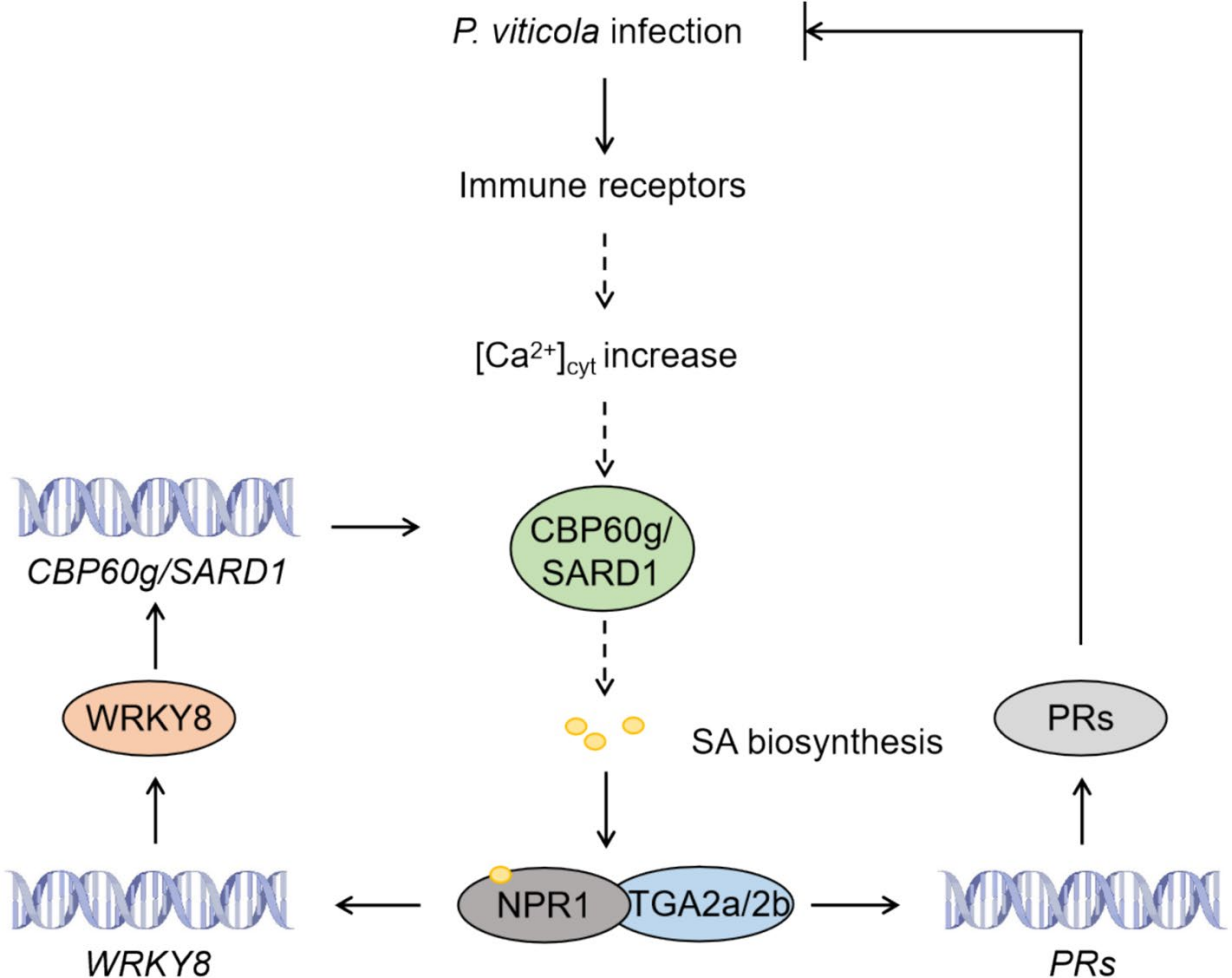
VvWRKY8 working model in *V. vinifera* resistance to *P. viticola*. Upon *P. viticola* infection, immune receptors on the plasma membrane recognize the pathogen, leading to cytosolic Ca^2^⁺ accumulation. Elevated [Ca^2^⁺]_cyt_ activates transcription of the genes encoding the CBP60 family transcription factors VvCBP60g and VvSARD1, promoting SA biosynthesis. Increased SA levels facilitate formation of a transcriptional regulator comprising the SA receptor VvNPR1 and the redundant transcription factors VvTGA2a and VvTGA2b, which activates SA-responsive target genes. This activation induces the expression of pathogenesis-related (PR) proteins that restrict *P. viticola* invasion and promotes accumulation of the WRKY transcription factor VvWRKY8. VvWRKY8 is recruited to the promoters of *VvCBP60g* and *VvSARD1*, and enhances their transcription. In turn, VvCBP60g and VvSARD1 amplify SA biosynthesis and further promote *VvWRKY8* transcription. Together, the CBP60g/SARD1-TGA2a/2b-WRKY8 module forms a positive feedback loop that enhances resistance to *P. viticola*

Genome analysis has identified 62 WRKY TFs in *V. vinifera* (Vodiasova et al. 2024), and several have been functionally characterized. For instance, VvWRKY5 upregulates the expression of *VvMYC2* and downregulates *VvJAZ2*, activating jasmonic acid (JA) signaling and thereby promoting grapevine resistance to white rot disease (Zhang et al. 2023). In *Vitis quinquangularis*, VqWRKY56 interacts with VqbZIPC22 and synergistically increases the transcript levels of *VvCHS3*, *VvLAR1*, and *VvANR*, promoting proanthocyanidin biosynthesis and enhancing resistance to powdery mildew (Wang et al. 2023a, b). In *V. vinifera*, VvWRKY18 upregulates *VvSTSs* (*Stilbene Synthase*) and interacts with VvNPR1, promoting stilbene accumulation and initiating systemic acquired resistance (SAR) (Wang et al. 2021). VviWRKY24 increases β-damascenone accumulation by directly upregulating *VviNCED1* and activating abscisic acid (ABA) signaling (Wei et al. 2025). In this study, VvWRKY8 acts as an SA signaling amplifier by upregulating *VvCBP60g* and *VvSARD1* and by being transcriptionally activated by VvTGA2a/2b (Fig. 8).

VvWRKY8, together with upstream regulators VvTGA2a/2b and downstream targets *VvCBP60g* and *VvSARD1*, promotes ROS accumulation (Figs. 3E, 5D, 7C). This result is consistent with previous reports that H₂O₂ facilitates the SA signaling pathway and the establishment of SAR (Chen et al. 1993; Cao et al. 2024). Positive feedback amplification of SA signaling involves NPR1 monomerization and upregulation of *ICS1*, *PBS3*, and *EDS5* (Ding et al. 2018; Sun et al. 2015). Our data suggest an additional mechanism in which VvWRKY8 links VvTGA2a/2b with *VvCBP60g* and *VvSARD1*, thereby amplifying SA biosynthesis and perception. We also considered the immune functions of the close homologs VvWRKY6 and VvWRKY47. Transcription of *VvWRKY8* was induced by SA and *P. viticola*, whereas *VvWRKY6* and *VvWRKY47* were not. These results indicate that VvWRKY8, rather than its homologs, plays a critical role in regulating grapevine immune responses to *P. viticola*.

Previous work showed that VvWRKY8 regulates the expression of multiple *VvSTSs* by interacting with VvMYB14, which is recruited to *VvSTSs* promoters and activates their expression (Jiang et al. 2019). VvWRKY8 restrains *VvSTSs* transcription by inhibiting the transcriptional activity of VvMYB14, thereby limiting resveratrol (Res) accumulation and helping maintain Res homeostasis in *V. vinifera* (Jiang et al. 2019). VvWRKY8 also interacts with VvMYB30, a repressor of *VvSTSs*, reinforcing this repression (Mu et al. 2023). Together, these findings suggest that VvWRKY8 modulates UV-induced Res synthesis through protein–protein interactions with VvMYB14 and VvMYB30 rather than through direct protein-DNA binding (Mu et al. 2023).

The *A. thaliana* homologs of VvWRKY8, AtWRKY45 and AtWRKY75, have also been studied. AtWRKY45 increases inorganic phosphate (Pi) uptake and content by directly upregulating *AtPHT1;1* (*Phosphate Transporter 1;1*) (Wang et al. 2014a, b). RNAi-mediated suppression of *AtWRKY75* reduces the expression of several Pi-starvation-induced genes, including phosphatases and high-affinity Pi transporters, resulting in increased susceptibility to Pi starvation (Devaiah et al. 2007). In addition, RNAi plants show increased lateral root length and number, as well as increased root hair number, compared with the wild type (Devaiah et al. 2007). The role of AtWRKY75 in root development was further supported by Rishmawi et al., who reported that AtWRKY75 regulates root hair number by modulating the expression of *CAPRICE*, which promotes root hair formation (Rishmawi et al. 2014). The apple homolog MdWRKY75 promotes thermotolerance by activating the expression of heat shock factor genes *MdHsfs* (Zhang et al. 2024).

Many studies indicate that homologues of VvWRKY8 function through hormone signaling pathways. AtWRKY75 enhances plant resistance to the necrotrophic pathogens *Botrytis cinerea* and *Alternaria brassicicola* by boosting jasmonic acid (JA) signaling (Chen et al. 2021a, b, c). AtWRKY75 binds to the promoter of *ORA59* (Octadecanoid-Responsive *Arabidopsis* 59), a JA-associated gene, and activates its expression (Chen et al. 2021a, b, c). AtWRKY75 is also involved in ABA-mediated leaf senescence and seed germination (Zhang et al. 2022). On the one hand, AtWRKY75 negatively regulates the expression of *GLK1* (Golden 2-Like 1) and *GLK2*, two repressors of ABA signaling. On the other hand, SIB1 (Sigma factor-binding protein 1) and SIB2 inhibit ABA-induced leaf senescence by interacting with AtWRKY75 and dampening its transcriptional activity (Zhang et al. 2022). AtWRKY75 accelerates flowering by directly activating expression of the flowering time integrator gene *FT* (*Flowering Locus T*) in a gibberellin (GA) signaling-dependent manner (Zhang et al. 2018). In contrast, the DELLA proteins RGL1 (Repressor of ga1-3 Like) and GAI (GA Insensitive) interact with AtWRKY75 and inhibit its transcriptional activity, thereby delaying flowering (Zhang et al. 2018). Similarly, AtWRKY45 directly activates the expression of several senescence-associated genes (SAGs), including *SAG12*, *SAG13*, and *SAG113*, and is under the control of RGL1 (Chen et al. 2017). DcWRKY75, another orthologue of VvWRKY8 in *Dianthus caryophyllus* L., upregulates the expression of two major ethylene biosynthetic genes, *DcACS1* [1-*Aminocyclopropane*−1-*Carboxylic Acid* (*ACC*) *Synthase* 1] and *DcACO1* (ACC oxidase) (Xu et al. 2021). Moreover, DcWRKY75 is regulated by DcEIL3-1 (Ethylene Insensitive 3 Like 3–1), which binds to the DcWRKY75 promoter and promotes its expression (Xu et al. 2021). AtWRKY75 also directly activates transcription of *AtSID2* (SA Induction Deficient 2; *ICS1*) while repressing transcription of *AtCAT2* (Catalase 2), forming a tripartite amplification loop that accelerates leaf senescence through SA and ROS signaling pathways (Guo et al. 2017). More recently, SlWRKY75 was reported to enhance tomato resistance to Pst DC3000 by regulating auxin biosynthesis homeostasis in cooperation with the repressor SlGH3.3 and the promoter of *SlVQ16* (Yang et al. 2024). In our study, we show that VvWRKY8 potentiates SA signaling through a distinct mechanism. VvWRKY8 directly initiates transcription of *VvCBP60g* and *VvSARD1*, and *VvWRKY8* expression is, in turn, promoted by VvTGA2a and VvTGA2b together with SA accumulation. Collectively, these findings indicate that VvWRKY8 and its homologues participate in diverse physiological and pathological processes in plants.

A sophisticated phytohormone network composed of auxin, GA, ethylene, ABA, SA, and JA signaling pathways acts synergistically and antagonistically to fine-tune plant growth, development, and resistance to biotic and abiotic stresses (Robert-Seilaniantz et al. 2011). To maintain stable operation of this network, key regulators coordinate hormonal crosstalk (Robert-Seilaniantz et al. 2011). The MYC family transcription factor MYC2 functions as a master regulator of JA signaling, governing the expression of numerous JA-responsive genes and interacting with JAZ (Jasmonate ZIM-domain) repressors (Chini et al. 2007; Cheng et al. 2011; Kazan and Manners 2013). The transcriptional activity of MYC2 is repressed by JAZ proteins under basal conditions but is released when JA accumulates in response to environmental stimuli (Kazan and Manners 2012). In addition, MYC2 activates expression of *RD22* (Responsive to Desiccation 22), an ABA responsive gene, thereby enhancing tolerance to drought stress (Abe et al. 2003). JA and SA signaling pathways are also known to function antagonistically in plant immunity. *ICS1* expression is elevated in the *nac* triple mutant lacking the NAC transcription factors ANAC019, ANAC055, and ANAC072 (Zheng et al. 2012). These NAC factors are directly activated by MYC2 (Zheng et al. 2012). Based on this evidence, VvWRKY8 and its homologues are induced by JA, ABA, GA, ethylene, auxin, and SA treatments and contribute to these signaling pathways (Chen et al. 2021a,b, c; Zhang et al. 2022; Zhang et al. 2018; Chen et al. 2017; Xu et al. 2021; Yang et al. 2024; Guo et al. 2017). Therefore, we hypothesize that VvWRKY8 and its homologues are important regulators of hormone crosstalk. Moreover, in addition to SA, VvWRKY8 may enhance pathogen resistance in grapevine by modulating other defense-related phytohormones, such as JA and ethylene, and by promoting resveratrol biosynthesis (Chen et al. 2021a, b, c; Xu et al. 2021; Jiang et al. 2019; Mu et al. 2023).

Overexpression of *VvWRKY8* triggers an ETI-related PCD response (Fig. 3D). However, overexpression of the upstream *VvTGA2a/2b* or the downstream *VvCBP60g* and *VvSARD1* cannot induce HR (Figure S5E). This suggests that *VvWRKY8* mediates the HR, but not in a completely SA-dependent manner. In addition to transcriptional regulation, WRKY8 is regulated by 26S proteasome-mediated protein degradation and by heterodimerization with MYB TFs (Jiang et al. 2019; Mu et al. 2023). In other words, the abundance and activity of the VvWRKY8 protein are tightly controlled in vivo, and overexpression of *VvWRKY8* or pathogen-derived effectors recognized by NLRs overcomes these constraints to elicit an ETI-related HR. In addition, *VvWRKY8*, *VvTGA2a/2b*, *VvCBP60g*, and *VvSARD1* promote ROS accumulation (Figs. 3E, 5D, and 7C), indicating that *VvWRKY8* enhances ROS production through SA signaling.

In this study, we show that *VvWRKY8*, an ortholog of *AtWRKY45* and *AtWRKY75* in *V. vinifera*, is induced by *P. viticola* inoculation and SA treatment. As a WRKY TF, VvWRKY8 localizes to the nucleus and displays transcriptional activity.Transient expression of *VvWRKY8* in grape leaves confers resistance to *P. viticola* through SA signaling, as SA-related genes are upregulated and SA accumulation increases. Further analyses reveal that *VvWRKY8* activates the expression of *VvCBP60g* and *VvSARD1*, two master regulators of the SA pathway, thereby enhancing SA signaling. In turn, *VvWRKY8* is an SA-responsive gene whose expression is directly upregulated by *VvTGA2a* and *VvTGA2b*, which bind the *VvWRKY8* promoter. Consistent with the effects of transient *VvWRKY8* expression, overexpression of *VvCBP60g*, *VvSARD1*, and *VvTGA2a/2b* also enhances grapevine resistance to *P. viticola*. We propose a positive feedback loop in SA signaling centered on *VvWRKY8* that contributes to defense against *P. viticola* invasion (Fig. 8).

## Materials and methods

### Plant materials and growth conditions

Grapevine (*Vitis vinifera* cv. ‘Thompson Seedless’ and ‘Cabernet Sauvignon’) and tobacco (*Nicotiana benthamiana*) were grown in a controlled growth chamber at 25 °C under a 16-h light/8-h dark photoperiod.

### Plasmid constructs

The full-length of coding sequences (CDSs) and promoter fragments of *VvWRKY8*, *VvCBP60g*, *VvSARD1*, *VvTGA2a*, and *VvTGA2b* were amplified by PCR and cloned into the indicated vectors using restriction enzyme digestion and ligation. 

The pHB-GFP vector was used for transient expression in planta; pB42AD and pLacZi were used for yeast one-hybrid (Y1H) assays; pGreenII 0800-LUC was used for dual-luciferase assays; pColdIII-GST and pET30a-His were used for recombinant protein expression; and pGBKT7 was used for yeast transcriptional activity assays.

All primers used for plasmid constructs are listed in Supplementary Table S1.

### Pathogen inoculation and salicylic acid treatment

*P. viticola* was propagated on detached leaves of ‘Thompson seedless’ or ‘Cabernet Sauvignon’ at room temperature. Sporangia were collected, suspended in sterile distilled water, and sprayed onto ‘Cabernet Sauvignon’ leaves. Infected leaves were were harvested at 0, 2, 6, 12, 24, 48 h post inoculation for RT-qPCR analysis.

For salicylic acid (SA) treatment, 100 μM SA was sprayed onto ‘Cabernet Sauvignon’ leaves, and samples were collected at the indicated time points for RT-qPCR analysis.

### Transient expression in plant

Agrobacterium tumefaciens strain GV3101 harboring pHB*-VvWRKY8-GFP,* pHB*-VvCBP60g-GFP,* pHB*-VvSARD1-GFP,* pHB*-VvTGA2a-GFP,* pHB*-VvTGA2b-GFP,* or the empty pHB*-GFP* was grown, collected by centrifugation, and resuspended in transformation buffer [10 mM MgCl_2_, 10 mM MES, 200 μM acetosyringone (AS), 0.03% Silwet L-77, pH = 5.8].

For grapevine transient expression, two-month-old ‘Thompson seedless’ plantlets were used. Grape leaves were immersed in the bacterial suspension and vacuum infiltratedat −3000 kPa for 5 min, repeated twice. After infiltration, leaves were rinsed with sterile distilled water and sampled 5 days later.

For tobacco, five-week-old tobacco plants were used. Bacterial suspensions were Infiltrated into leaves using a needleless syringe, and samples were collected 2 days after infilitration.

### *P. viticola* inoculation assay

Grape leaves were excised into discs using a perforator and placed on moist filter paper in Petri dishes. A suspension of *P. viticola* sporangia was applied to the leaf discs. At 7 days post inoculation, sporangia were washed off with sterile distilled water and quantified using a hemocytometer.

### RT-qPCR

Total RNA of grape leaves was extracted using CTAB-based method as described previously (Reid et al*.* 2006). The first strand cDNA was synthesized using the Hifair III 1st strand cDNA Synthesis SuperMix for qPCR (gDNA digester plus) Kit (YEASEN). RT-qRCR were performed on C100 Touch Thermal Cycler (BIO-RAD) using Hieff qPCR SYBR Green Master Mix (YEASEN). All primers used for RT-qPCR assay are listed in Supplementary Table S2.

### Histochemical staining

For aniline blue staining, leaf discs were decolorized in saturated chloral hydrate for 2 days and then stained in 0.05%(w/v) aniline blue solution.

For trypan blue staining, leaf discs were immersed in trypan blue solution (0.25% w/v, prepared in a mixture of ethanol:glycerol:phenol:water at equal volumes), boiled for 5 min, and decolorized insaturated chloral hydrate.

For DAB staining, leaf discs were infiltrated in DAB solution (0.1% w/v in Tris–HCl; pH = 3.8) under vacuumat −3000 kPa for 5 min, repeated twice, and incubated in the dark for 24 h. Samples were then decolorized in ethanol:glycerol:acetic acid (3:1:1, v/v/v) by boiling for 10 min.

### Determination of SA content

Grape leaves were taken and ground into powder in liquid nitrogen. Approximately 100 mg of tissue was extracted overnight −20 °C in methanol: isopropanol(1: 5) by vertexing. After centrifugation at 1,2000 g for 10 min at 4 °C, the supernatant was collected and dried under nitrogen stream. The residue was resuspended in 200 μL resuspend solution (0.2 M sodium acetate, pH 5.5, methanol 9: 1, v/v) and incubated at 4 °C for 1 h. 12 units β-Glucosidase was added, followed by incubation at 37 °C for 90 min and enzyme inactivation at 85 °C for 5 min. After centrifugation at 1,2000 g for 5 min, the supernatant was filtrated and subjected to HPLC analysis.

SA was quantified using a Waters e2695 Separations Module coupled with a Waters 2475 Multi λ Fluorescence Detector (alliance). Flow phase A is 0.2 M sodium acetate (pH 5.5) and Flow phase B is methanol. The flow rate is 0.8 mL per minutes. The excitation and emission wavelengths were set to 305 nm and 407 nm, respectively.

### Yeast one-hybrid (Y1H) assay

Yeast one-hybrid assays were performed using the yeast strain EGY48. The indicated bait (pLacZi derivatives) and prey (pB42AD derivatives) constructs were co-transformed into yeast cells. Transformants were selected on SD/− Trp/− Ura medium and further screened on SD/− Trp/− Ura medium supplemented with galactose, raffinose, and 80 mg mL⁻^1^ X-gal. 

### Dual-Luciferase assay

Effector vectors pHB*-VvWRKY8-GFP*, pHB*-VvTGA2a-GFP*, pHB*-VvTGA2b-GFP*, or pHB*-GFP* were transformed into *Agrobacterium tumefaciens* strain GV3101, while reporter vectors pGreenII*-proVvCBP60g,* pGreenII*-proVvSARD1,* or pGreenII*-proVvWRKY8* were transformed into GV3101 with helper. Equal volume of effector strain and reporter strain were co-infiltrated into tobacco. Leaf samples were collected 2 days after infiltration, and luciferase activity was measured using the Dual-Luciferase Reporter Assay Kit (YEASEN) on a GloMax Multi Detection System (Promega).

### Electrophoretic mobility shift assay (EMSA)

Recombinant GST- or His-tagged proteins were expressed in *Escherichia coli* strain BL21 andpurified using affinity agarose beads.

Biotin-labeled 25 bps DNA probes corresponding to promoter fragments of *VvWRKY8*, *VvCBP60g*, and *VvSARD1* were synthesized. EMSA was performed using Light-Shift Chemiluminescent EMSA Kit (Thermo Scientific) according to the manufacturer’s instructions.

## Supplementary Information

Additional file 1: Supplementary Fig. S1. Transcriptome analysis implies that TF VvWRKY8 functions in grape response to *P. viticola*. Supplementary Fig. S2. *VvWRKY8* and its homologies Supplementary Fig. S3. Identities of *VvWRKY8* expression. Supplementary Fig. S4. *VvCBP60g* and *VvSARD1* are the candidate target genes of VvWRKY8. Supplementary Fig. S5. VvCBP60g and VvSARD1 play roles in grape immunity. Supplementary Fig. S6. *VvWRKY8* might be the downstream target of VvTGA2a/2b. Supplementary Fig. S7. Expression patterns of *VvTGA2a/2b* induced by *P. viticola* and SA. Supplementary Fig. S8. VvTGA2a/2b positively regulate grape defense to *P. viticola*. Supplementary Fig. S9. VvTGA2a and VvTGA2b interact with VvNPR1 and VvNPR3. Supplementary Fig. S10. RNAi assay. Table S1 Primers used for constructing vectors. Table S2 Primers used for RT-qPCR. Table S3 DNA probes used for EMSA assay.

Additional file 2. Data 1. Reads of genes in transcriptome and WGCNA analysis. Data 2. KEGG enrichment analysis of different modules. Data 3. DEGs analysis (II). Data 4. DEGs analysis (CI). Data 5. DEGs analysis (II vs CI). Data 6. Transcription factors in transcriptome. Data 7. Venn diagram. Data 8. Motif enrichment analysis. Data 9. Reads of genes in transcriptome.

## Data Availability

Data will be available from the corresponding author upon reasonable request.

## Abbreviations

AS: Acetosyringone
BiFC: Bimolecular fluorescence complementation
CDS: Coding sequence
CTAB: Cetyltrimethylammonium bromide
DAB: Diaminobenzidine
EMSA: Electrophoretic mobility shift assay
GFP: Green fluorescent protein
HPLC: High performance liquid chromatography
RT-qPCR: Real-time quantitative PCR
SA: Salicylic acid
TF: Transcription factor
X-gal: 5-Bromo-4-chloro-3-indolyl-β-D-galactopyranoside
α-gal: 5-Bromo-4-chloro-3-indoxyl-α-D-galactopyranoside
Y1H: Yeast one-hybrid
Y2H: Yeast two-hybrid
