# Supplementary material for: A VvWRKY8-salicylic acid pathway amplification loop confers resistance to Plasmopara viticola in grapevine
Source: Mol Hortic. 2026 Aug 5;6:61. doi: 10.1186/s43897-026-00233-y (PMC13440096; doi:10.1186/s43897-026-00233-y)
Supplement: Supplementary file 2 [file 43897_2026_233_MOESM1_ESM.doc]

Supplementary **Fig. S1** Transcriptome analysis implies that TF VvWRKY8 functions in grape response to *P. viticola*. A *P. viticola* isolates *Pv*18002 and *Pv*18005 were inoculated in four Eurasian grapevine cultivars Cabernet Sauvignon, Thompson Seedless, Chardonnay and Rizamat, respectively. Pictures were taken at seven days post inoculation (dpi). **B** *P. viticola* sporangia counting. The number of *P. viticola* sporangia on each grape leaf disc inoculated with *Pv*18002 (CI) or *Pv*18005 (II) was counted at 7 dpi. The error bars represent the SE (n=4; ****, p value < 0.0001 in student’s t-test). **C** and **D** DEGs numbers between 0 hpi and each time point in CI group (**C**) and II group (**D**). E Transcriptional activity assay. Coding sequences (CDS) of *VvWRKY8* and *VvWRKY12* were expressed in yeast cells. SD/-Trp medium selected positive transformants, and transcriptional activity was assessed on SD/-Trp/-His medium supplemented with α-gal.

Supplementary **Fig. S2** *VvWRKY8* and its homologs. **A** Amino acid sequence alignment of VvWRKY8 and its homologies in *V. vinifera*. **B** to **D** Expression patterns of *VvWRKY8* (**B**),*VvWRKY6* (**C**) and *VvWRKY47* (**D**) in transcriptome. Reads values represented expression levels and were standardized to the value at 0 hpi. The error bars mean SE (n=3). ns, no significance; *, p value < 0.05 (two-way ANOVA and Sidack’s tests). **E** to **G** RT-qPCR analysis of VvWRKY6 (**E**), VvWRKY47 (**F**) and *VvPR1* (**G**) in ‘Cabernet Sauvignon’ grape leaves after 100 µM SA treatment at different time points. *VvGAPDH* served as internal reference. Expression levels at 0 hpi were set to 1. The error bars mean the SE (n=3).

Supplementary **Fig. S3** Verification of VvWRKY8 expression in grape leaves. **A** Fluorescence signals of GFP and VvWRKY8-GFP proteins in ‘Thompson Seedless’ grape leaves at 5 days after Agrobacterium-mediated transformation. Images were captured using confocal microscopy. Scale bar is 50 µm. **B** RT-qPCR analysis of VvWRKY8 transcript levels in grape leaves at 5 days after transformation. Expression levels were normalized to those in GFP-expressing control leaves, with *VvGAPDH* as internal reference. Error bars represent SE of three biological replicates. *, p < 0.05 (Student's t-test).

Supplementary **Fig. S4** *VvCBP60g* and *VvSARD1* are candidate target genes of VvWRKY8. **A** and **B** RT-qPCR-based transcriptional profilings of *VvCBP60g* (**A**) and *VvSARD1* (**B**) in ‘Cabernet Sauvignon’ grape leaves after *P. viticola* isolate *Pv*18002 infection. *VvGAPDH* gene was used as internal reference. Expression levels at 0 hpi were set to 1. ns, no significance; *, p value < 0.05; **, p value <0.01; ***, p value<0.001 (two-way ANOVA and Sidack’s tests). The error bar means the SE of three biological replicates. **C** and **D** Westernblot analyses of GFP and VvWRKY8-GFP in *N. benthamiana* leaves in dual-luciferase assays (Figure 4, D and E).

Supplementary **Fig. S5** VvCBP60g and VvSARD1 play roles in grape immunity. **A** and **B** Transcriptional expression patterns of *VvCBP60g* (**A**) and *VvSARD1* (**B**) under SA treatment. Grape leaves were treated with 100 µM SA and collected in 0, 2, 6, 12, 24 and 48 hours respectively. *VvGAPDH* served as internal control. Expression levels at 0 hpi were set to 1. The error bar means the SE of three biological replicates. ns, no significance; *, p value < 0.05; **, p value <0.01 (two-way ANOVA and Sidack’s tests). **C** Fluorescent signals indicate the expressions of target protein. Pictures were taken at five days after transformation in *V. vinifera*. Scale bars are 50 µm. **D** Subcellular localizations of VvCBP60g and VvSARD1. Pictures were taken at two days after transformation in *N. benthamiana*. Scale bars represent 20 µm. **E** Trypan blue staining of grape leaves expressing GFP, VvCBP60g-GFP and VvSARD1-GFP proteins. Assays were conducted at five days after transformation.

Supplementary **Fig. S6** VvWRKY8 is a potential downstream target of VvTGA2a/2b. **A** Phylogenetic analysis of TGA family members in *V. vinifera* and *A. thaliana*. The phylogenetic tree was constructed using the neighbor-Joining method with 1,000 bootstrap replicates. Branch lengths in the same units indicate the evolutionary distances. **B** Western blot analysis of GFP, VvTGA2a-GFP and VvTGA2b-GFP in *N. benthamiana* leaves in dual-luciferase assays. Samples were collected at two days after transformation.

Supplementary **Fig. S7** Expression patterns of VvTGA2a/2b induced by *P. viticola* and SA. **A** and **B** *P. viticola* induced transcription expression patterns of *VvTGA2a* (**A**) and *VvTGA2b* (**B**) in *V. vinifera*. **C** and **D** RT-qPCR-based transcriptional profiles of *VvTGA2a* (**C**) and *VvTGA2b* (**D**) after SA treatment in *V. vinifera*. Grape leaves were sampled in 0, 2, 6, 12, 24 and 48 hours after *P. viticola* or SA treatment. Expression levels at 0 hpi under *P. viticola* or SA treatment were set as 1. *VvGAPDH* was used as internal reference. Error bars represent SE (n=3). ns, no significance; *, p value < 0.05 (two-way ANOVA and Sidack’s tests).

Supplementary **Fig. S8** VvTGA2a/2b positively regulate grape defense against *P. viticola*. **A** Transcriptional activity analyses of VvTGA2a/2b and VvNPR1/3. Recombined pGBKT7 vectors carrying the CDS of indicated genes were transformed into yeast strain AH109 cells. Positive transformants growing on SD/-Trp medium were transplanted to SD/-Trp/-His/+α-gal medium. Pictures were taken at three days after transplant. **B** Subcellular localizations of VvTGA2a and VvTGA2b. Two days after agrobacterium-mediated transformation in tobacco leaves, pictures were taken with confocal microscope. Scale bars represent 20 µm. **C** and **D** RT-qPCR analyses for transcription expression levels of VvTGA2a (**C**) and VvTGA2b (**D**). Expression levels of each gene under GFP control were set to 1. *VvGAPDH* was used as internal reference. Error bars represent SE (n=3). ns, no significance; *, p-value < 0.05; **, p-value < 0.01; ****, p value < 0.0001 (t-test). **E** Fluorescent signal indicated VvTGA2a/2b-GFP proteins. Pictures were taken in five days after transformation. Scale bars are 50 µm.

Supplementary **Fig. S9** VvTGA2a and VvTGA2b interact with VvNPR1 and VvNPR3. **A** Y2H assay of the interaction between VvTGA2a/2b and VvNPR1/3. Original and recombined vectors were co-transformed into yeast strain AH109 as indicated. Positive transformants on SD/-Trp/-Leu medium were transferred to SD/-Trp/-Leu/-His medium and after three days pictures were taken. **B** BiFC assay suggests that VvTGA2a/2b bind to VvNPR1/3. Fluorescence signals were detected in two days after transformation with confocal microscope in *N. benthamiana*. Scale bars are 20 µm.

Supplementary **Fig. S10** RNAi assay. **A** Schematic presentations of the vectors used in RNAi assay. CDS sequences and reverse complementary (rc) sequences were fused into the vector. **B** RT-qPCR assay revealed the expression levels of *VvWRKY8*. Expression levels of *VvWRKY8* in siGUS group were set to 1. *VvGAPDH* was used as internal reference. Error bars represent SE (n=3). ns means no significance in t-test.

**Table S1 Primers used for constructing vectors**

| Vector | Gene name | DNA sequence | |
| --- | --- | --- | --- |
| p*HB-GFP* | *VvWRKY8* | F | CGGGATCC ATGGATAGCTTCTCCACTCTC |
|  |  | R | GCACTAGT AAAGGAAGCATAAACTTGCATC |
|  | *VvCBP60g* | F | CGGGATCC ATGGAGAGTTTGGCATCTCTC |
|  |  | R | GCACTAGT ATCCCAAATCGGGGTTGAGCAG |
|  | *VvSARD1* | F | CGGGATCC ATGGCAGCTAAACGGCTTTTTG |
|  |  | R | GCACTAGT ACATGGGAAATGCTTAGAAG |
|  | *VvTGA2a* | F | CGGGATCC ATGGGGAGTATAGCAGTGAG |
|  |  | R | GCACTAGT CTCTCTTGGACGTGCCAGCC |
|  | *VvTGA2b* | F | CGGGATCC ATGGGTAGTAGAACAGTGAAG |
|  |  | R | GCACTAGT TTCCCGTGGCCGCGCAAGCC |
| p*LacZi*  and p*GreenII-0800* | *proCBP60g* | F | GGGGTACC AAGATTGCATTCCTAAGTTC |
|  |  | R | ACGCGTCGAC CATAATTCCAAAACTCTAG |
|  | *proSARD1* | F | GGGGTACC TACATCACACGCTCATCATC |
|  |  | R | ACGCGTCGAC CATCTTTCAAGAAATTCAAC |
|  | *proVvWRKY8* | F | GGGGTACC ATGTTCTGAAACGAGGTTG |
|  |  | R | ACGCGTCGAC CATGGATAGAAAATTAACAG |
| p*B42AD* | *VvWRKY8* | F | CCGGAATTC ATGGATAGCTTCTCCACTCTC |
|  |  | R | CCGCTCGAG AAAGGAAGCATAAACTTGCATC |
|  | *VvTGA2a* | F | CCGGAATTC ATGGGGAGTATAGCAGTGAG |
|  |  | R | CCGCTCGAG CTCTCTTGGACGTGCCAGCC |
|  | *VvTGA2b* | F | CCGGAATTC ATGGGTAGTAGAACAGTGAAG |
|  |  | R | CCGCTCGAG TTCCCGTGGCCGCGCAAGCC |
| p*ColdIII-GST* | *VvWRKY8* | F | CCGGAATTC ATGGATAGCTTCTCCACTCTC |
|  |  | R | GCGTCGAC AAAGGAAGCATAAACTTGCATC |
| p*ET30a-His* | *VvTGA2a* | F | CCGGAATTC ATGGGGAGTATAGCAGTGAG |
|  |  | R | GCGTCGAC CTCTCTTGGACGTGCCAGCC |
|  | *VvTGA2b* | F | CCGGAATTC ATGGGTAGTAGAACAGTGAAG |
|  |  | R | GCGTCGAC TTCCCGTGGCCGCGCAAGCC |
| p*GBKT7* | *VvWRKY8* | F | CCGGAATTC ATGGATAGCTTCTCCACTCTC |
|  |  | R | GCGTCGAC AAAGGAAGCATAAACTTGCATC |
|  | *VP16AD* | F | CCGGAATTC ATGGCCCCCCCGACCGATG |
|  |  | R | GCGTCGAC CCCACCGTACTCATCAATTC |
|  | *AtHY5* | F | CCGGAATTC ATGCAGGAACAAGCGACTAG |
|  |  | R | GCGTCGAC AAGGCTT GCATCAGCATTAG |
|  | *VvTGA2a* | F | CCGGAATTC ATGGGGAGTATAGCAGTGAG |
|  |  | R | GCGTCGAC CTCTCTTGGACGTGCCAGCC |
|  | *VvTGA2b* | F | CCGGAATTC ATGGGTAGTAGAACAGTGAAG |
|  |  | R | GCGTCGAC TTCCCGTGGCCGCGCAAGCC |
|  | *VvNPR1* | F | CCGGAATTC ATGGACTACAGAGCTGCTCTC |
|  |  | R | GCGTCGAC CTTCTTGCAAGAGAGTCTAC |
|  | *VvNPR3* | F | CCGGAATTC ATGGCCAATTCAGCTGAGCC |
|  |  | R | GCGTCGAC TAATTTTCTAGCCTTGTGAC |
| *pHellsgate 12* | *VvWRKY8* | F | GAAGGAGCCCTTCACCGGATCCATGGATAGCTTCTCCACTC |
|  |  | R | CGGGTTTGAGCTCAAAGAATTCAGCATATCGGGGCTTTCTAATC |

**Table S2 Primers used for RT-qPCR**

| Gene name | DNA sequence | |
| --- | --- | --- |
| *VvWRKY8* | F | AAACCAGGAGCCAAGTAGAT |
|  | R | CCACGATTCCTTCGTCTTTT |
| *VvCBP60g* | F | TGGCATCTCTCAAAAGGTGT |
|  | R | AGGGCAATTTACTGGCAAAA |
| *VvSARD1* | F | GGCATTTAGGGGGAATTCAT |
|  | R | GGGTTGTTTTCAGCATCTCT |
| *VvTGA2a* | F | CTGACTTTGGGACACTTGAG |
|  | R | GACTCTCCCCAGTTCTCTAC |
| *VvTGA2b* | F | AATCTCTGGCTGAGACGTTA |
|  | R | GAAGTCGGGAGAAGTAGTCA |
| *VvPR1* | F | ATGTTTGTGTACGATGCTATTGATATTCC |
|  | R | ATAAGGACGTTGTCCGATATAGTTGCC |
| *VvPR2* | F | AGAAGCCCAAATAGGAGTGT |
|  | R | ATATCTTGAAGGGCGTTGTT |
| *VvPR4* | F | GGGCCACCTACCATTACTAT |
|  | R | GTTTGTCACACTAAGGCACT |
| *VvPR5* | F | ATGATAGTGGAAGGGAGTGG |
|  | R | CACATGTGAAGGTACTGGTG |
| *VvICS1* | F | TGTCTTCTCTTCACCCAACT |
|  | R | GCTCCTCCCACTCTAAAGAT |
| *VvGAPDH* | F | GCCATGTGGACAATCAAG |
|  | R | AAGGAGGAGTCAGAGGGA |

**Table S3 DNA probes used for EMSA assay**

| Promoter | Probe name | DNA sequence | | Biotin labeled |
| --- | --- | --- | --- | --- |
| *proVvWRKY8* | Probe | F | ATAAATCGAGTGACGACTTTGAGGA | yes |
|  |  | R | TCCTCAAAGTCGTCACTCGATTTAT | no |
|  | mProbe | F | ATAAATCGAGTACAAACTTTGAGGA | yes |
|  |  | R | TCCTCAAAGTTTGTACTCGATTTAT | no |
|  | Competitor | F | ATAAATCGAGTGACGACTTTGAGGA | no |
|  |  | R | TCCTCAAAGTCGTCACTCGATTTAT | no |
| *proVvCBP60g* | Probe | F | ATTAGCAAATTGACCCCAATTCGAC | yes |
|  |  | R | GTCGAATTGGGGTCAATTTGCTAAT | no |
|  | mProbe | F | ATTAGCAAATGGGTCCCAATTCGAC | yes |
|  |  | R | GTCGAATTGGGACCCATTTGCTAAT | no |
|  | Competitor | F | ATTAGCAAATTGACCCCAATTCGAC | no |
|  |  | R | GTCGAATTGGGGTCAATTTGCTAAT | no |
| *proVvSARD1* | Probe | F | GAGAGAATTTTGACCTTTTAGAGTA | yes |
|  |  | R | TACTCTAAAAGGTCAAAATTCTCTC | no |
|  | mProbe | F | GAGAGAATTTGGGTCTTTTAGAGTA | yes |
|  |  | R | TACTCTAAAAGACCCAAATTCTCTC | no |
|  | Competitor | F | GAGAGAATTTTGACCTTTTAGAGTA | no |
|  |  | R | TACTCTAAAAGGTCAAAATTCTCTC | no |
